# Developing sero-diagnostic tests to facilitate *Plasmodium vivax* Serological Test-and-Treat approaches: modeling the balance between public health impact and overtreatment

**DOI:** 10.1186/s12916-022-02285-5

**Published:** 2022-03-18

**Authors:** Thomas Obadia, Narimane Nekkab, Leanne J. Robinson, Chris Drakeley, Ivo Mueller, Michael T. White

**Affiliations:** 1 G5 Épidémiologie et Analyse des Maladies Infectieuses, Département de Santé Globale, Institut Pasteur, F-75015 Paris, France; 2 Unité Malaria: Parasites et Hôtes, Département Parasites et Insectes vecteurs, Institut Pasteur, F-75015 Paris, France; 3 Hub de Bioinformatique et Biostatistique, Département de Biologie Computationnelle, Institut Pasteur, F-75015 Paris, France; 4 Swiss Tropical and Public Health Institute, Basel, Switzerland; 5 University of Basel, Basel, Switzerland; 6 Population Health & Immunity Division, Walter and Eliza Hall Institute of Medical Research, Parkville, Australia; 7 Department of Medical Biology, University of Melbourne, Melbourne, Australia; 8 Burnet Institute, Melbourne, Victoria Australia; 9 Department of Immunology and Infection, London School of Hygiene and Tropical Medicine, London, UK

**Keywords:** *Plasmodium vivax* malaria, Test development, Serological test-and-treat, Modeling, Public health interventions, Overtreatment

## Abstract

**Background:**

Eliminating *Plasmodium vivax* will require targeting the hidden liver-stage reservoir of hypnozoites. This necessitates new interventions balancing the benefit of reducing *vivax* transmission against the risk of over-treating some individuals with drugs which may induce haemolysis. By measuring antibodies to a panel of *vivax* antigens, a strategy of serological-testing-and-treatment (*Pv*SeroTAT) can identify individuals with recent blood-stage infections who are likely to carry hypnozoites and target them for radical cure. This provides a potential solution to selectively treat the *vivax* reservoir with 8-aminoquinolines.

**Methods:**

*Pv*SeroTAT can identify likely hypnozoite carriers with ~80% sensitivity and specificity. Diagnostic test sensitivities and specificities ranging 50–100% were incorporated into a mathematical model of *vivax* transmission to explore how they affect the risks and benefits of different *Pv*SeroTAT strategies involving hypnozoiticidal regimens. Risk was measured as the rate of overtreatment and benefit as reduction of community-level *vivax* transmission.

**Results:**

Across a wide range of combinations of diagnostic sensitivity and specificity, *Pv*SeroTAT was substantially more effective than bloodstage mass screen and treat strategies and only marginally less effective than mass drug administration. The key test characteristic determining of the benefit of *Pv*SeroTAT strategies is diagnostic sensitivity, with higher values leading to more hypnozoite carriers effectively treated and greater reductions in *vivax* transmission. The key determinant of risk is diagnostic specificity: higher specificity ensures that a lower proportion of uninfected individuals are unnecessarily treated with primaquine. These relationships are maintained in both moderate and low transmission settings (qPCR prevalence 10% and 2%). Increased treatment efficacy and adherence can partially compensate for lower test performance. Multiple rounds of *Pv*SeroTAT with a lower performing test may lead to similar or higher reductions in *vivax* transmission than fewer rounds with a higher performing test, albeit with higher rate of overtreatment.

**Conclusions:**

At current performance, *Pv*SeroTAT is predicted to be a safe and efficacious option for targeting the hypnozoite reservoir towards *vivax* elimination. *P. vivax* sero-diagnostic tests should aim for both high performance and ease of use in the field. The target product profiles informing such development should thus reflect the trade-offs between impact, overtreatment, and ease of programmatic implementation.

**Supplementary Information:**

The online version contains supplementary material available at 10.1186/s12916-022-02285-5.

## Background

*Plasmodium vivax* remains a major cause of malaria [1, 2], with its importance, relative to *P. falciparum*, increasing as case management and conventional vector-control measures such as long-lasting insecticidal bednets reduce transmission to low levels [3]. This difficulty in achieving pre-elimination levels of *P. vivax* transmission is largely attributed to the parasite’s particular biology, with long-lasting liver-stage hypnozoites causing blood-stage relapses weeks to years following the initial infection. These relapses are thought to account for up to 80% of all detected *P. vivax* bloodstage infections [4, 5] and contribute to sustained malaria transmission. These hypnozoites form a hidden parasite reservoir that needs to be targeted in order to effectively reduce the *vivax* malaria burden.

The current mainstay of *P. vivax* control is treatment with 8-aminoquinolines such as primaquine or tafenoquine, the only drugs effective at clearing hypnozoites. However, their administration requires careful medical assessment due to the risk of potentially inducing severe hemolysis in patients with glucose-6-phosphate dehydrogenase deficiency (G6PDd), an X-linked genetic disorder affecting ~5% of the population in malaria-endemic regions with geographical variations [6–8]. G6PDd may be diagnosed either by point-of-care rapid diagnostic tests or using quantitative laboratory assays. Primaquine efficacy is affected by dosage and patient adherence. The duration and dose of a primaquine regimen vary greatly in both duration of treatment (7 or 14 days) and total dose (3.5–7mg/kg) [9]. Poor adherence is regularly reported [10] which may substantially undermine the efficacy of radical cure of *vivax* malaria, resulting in the development of short-course high-dose primaquine regimens [11]. In recent clinical trials, tafenoquine has shown promising results with comparable efficacy to low-dose primaquine (3.5mg/kg total dose) in preventing relapses with only a single dose [12]. While a patient’s G6PDd status affects eligibility for 8-aminoquinline treatment, the efficacy of these drugs can also be altered by insufficient dosing or low CYP2D6 metabolization, for which treatment failures have been observed previously [13, 14].

For the treatment of individuals with symptomatic *P. vivax* infection, the risk of prescribing 8-aminoquinolines needs to be balanced against the benefit to the patient, accounting for the high probability that they carry hypnozoites and will experience future relapses with associated chronic morbidity and sustain onward. For public health interventions where entire populations are targeted for treatment with 8-aminoquinolines, the balance between benefit and risk is different, given that a large proportion of individuals will not be hypnozoite carriers. One strategy is mass drug administration (MDA) where all individuals are offered treatment, subject only to some basic eligibility criteria (e.g., age, pregnancy status). MDA with 8-aminoquinolines has been predicted to be highly effective in aiding *P. vivax* control and elimination [4, 15]. However, given the high prevalence of G6PDd in most malaria endemic settings, MDA with 8-aminoquinolines without prior G6PD testing would expose many individuals (including those without hypnozoites) to potentially dangerous drugs and is thus considered unsafe in most remaining *P. vivax* endemic areas. As a consequence, WHO does not currently recommend the use of MDA with 8-aminoquinolines for *P. vivax* malaria [16]. An alternative to MDA is mass screen and treat (MSAT) where individuals are first tested for blood-stage parasites with a rapid diagnostic test (RDT) or light microscopy, and only individuals with detectable blood-stage parasites are treated. MSAT with blood-stage drugs and primaquine has been shown not to have an effect on *P. vivax* transmission [17]. This is thought to be because a high proportion of *P. vivax* blood-stage infections and all *P. vivax* liver-stage infections are not detectable by RDTs or light microscopy.

The existing options for population-level interventions that are based on treatment with 8-aminoquinolines either lack impact (MSAT) or expose populations to a high degree of risk (MDA). By measuring antibodies to a panel of *P. vivax* antigens, it is possible to identify individuals with recent blood-stage infections who are likely to carry hypnozoites and target them for treatment with 8-aminoquinolines [18]. *P. vivax* serological test and treat (*Pv*SeroTAT), where people are screened by a serological test and, if classified as exposed, treated with a combination blood- and liver-stage treatment could thus provide a potentially safe and efficacious alternative for population-level treatment (see malaria serology use case 5 in Greenhouse *et al.* [19]).

Future programmatic implementation of *Pv*SeroTAT will depend on having effective, field-deployable *P. vivax* sero-diagnostic tests (SDTs). As with any diagnostic test, a *P. vivax* SDT will have to trade off sensitivity and specificity, and thus, the ability to detect all true positives requiring treatment while limiting overtreatment. The current diagnostic performance of these serological markers is approximately 80% sensitivity and 80% specificity [18]. Here, we model a wider range of test performance values (50–100%) and explore the relationship between the *P. vivax* SDT performance and *Pv*SeroTAT public health impact as well as overtreatment in order to inform *P. vivax* SDT target product profiles (TPPs). The mathematical model of *vivax* transmission from White *et al.* [20, 21] was adapted to implement *Pv*SeroTAT campaigns at the population level. We explored the range of possible sensitivities and specificities of this serological diagnostic tool and evaluated the corresponding drop in *P. vivax* prevalence after evaluating different *Pv*SeroTAT scenarios. These were compared to those potentially achievable in MDA or MSAT campaigns.

## Methods

### P. vivax transmission model

A previously developed *P. vivax* transmission model [21] was modified to simulate the impact of population-based testing and treatment strategies. Humans are represented in an individual-based, compartmental framework that models the particular biology of *P. vivax* infections. Mosquito population dynamics are modeled using a compartmental model. As the model was calibrated to replicate the transmission observed in Papua New-Guinea, its output represents a country with endemic *vivax* malaria where transmission is mostly peri-domestic.

Primaquine treatment and testing for G6PDd were simulated using a treatment pathway model accounting for primaquine eligibility, adherence, and overall effectiveness as per Nekkab *et al*. [22]. Briefly, any individual testing positive for recent *vivax* exposure undergoes a decision tree with respect to primaquine eligibility that depends on age, gender, pregnancy status, and G6PD activity. These treatment pathways applied both for routine case-management and public-health interventions.

### Demographics, G6PD deficiency, and CYP2D6 slow metabolizers

Population demographics were calibrated to reflect that of individuals found in *vivax* endemic areas such as PNG, with young populations (mean age 22.5 years) [21]. The prevalence of G6PDd was fixed at 4.6% as observed in a recent clinical trial conducted in the neighboring Solomon Islands (data not shown), consistent with reports from other studies of ~4.5% prevalence of G6PDd in Melanesia [6]. To that aim, individual levels of G6PD activity scores were sampled from sex-specific distributions. Prevalence of low CYP2D6 metabolism was chosen at 3.4% of the population, as observed in the same clinical trial.

### Model simulations

We modeled sustained malaria transmission in a population of size 20,000 over a period of 30 years, with daily time increments where individuals get exposed to mosquitoes and may become infected with *P. vivax*. Transmission was assumed to be perennial and non-seasonal so that seasonal effects did not confound evaluation of the effectiveness of simulated interventions. Two transmission settings were investigated, a low transmission setting (qPCR prevalence ~2%) and a moderate one (qPCR prevalence ~10%).

Case-management at the start of the simulation assumed that primaquine was routinely administered for patients treated after a symptomatic *P. vivax* infection, so that blood-stage and liver-stage parasites could be cleared with efficacy depending on modeled scenario (see below). Single or multiple rounds of interventions were evaluated, with up to three intervention rounds each separated with a 6-month period.

### Intervention parameters and outcome measures

Three population-level treatment strategies were simulated: (i) MDA, (ii) MSAT with light microscopy detection of blood-stage parasitemia, and (iii) *Pv*SeroTAT. All intervention campaigns assumed a base coverage of 80%. For *Pv*SeroTAT, interventions were simulated under all combinations of *P. vivax* SDT sensitivity and specificity ranging 50–100% by steps of 2.5%. Our current serological assay is described in detail by Longley *et al*. [18] This serological assay includes 8 *P. vivax* proteins including RBP2b_161-1454_ (PVX_094255) and MSP1-19 (PVX_099980). It is anticipated that optimization of the existing panel of proteins will lead to improved diagnostic sensitivity and specificity.

Three main scenarios were considered*: (i)* a “best-case” setting, where a hypothetical drug with no contra-indication achieved 100% efficacy in clearing liver-stages of the parasite regardless of metabolic disorders and where drug accessibility was never an issue; *(ii)* a high efficacy, high adherence setting where we modeled the effect of an 8-aminoquinoline comparable to the high dose, short-course primaquine course administered in the IMPROV study [11]; and (*iii)* a “real-life” setting corresponding to the situation in Brazil where primaquine is routinely prescribed, and where there is good quality data available on adherence to treatment, case-management and drug efficacy. For every combination of parameters, 100 stochastic simulations were conducted and results were averaged across replicates. A summary of relevant model parameters along with their values is provided in Table 1.

**Table 1:** Summary of drug accessibility, adherence, efficacy, and counter-indications across all three envisioned scenarios. The “best-case” scenario corresponds to a hypothetical drug that achieves 100% clearance of liver-stage parasites and does not pose any threat to health regardless of various deficiencies. The “High efficacy” scenario presents parameters corresponding to those observed in a clinical trial with administration of high-dose primaquine (7mg/kg total dose administered over 7 days) to clear liver-stages in eligible patients [11]. The “Real-life” scenario depicts the typical parameter values observed in Brazil where primaquine (3.5mg/kg total dose administered over 7 days) is routinely administered for *P. vivax*

	Best-case	High efficacy	Real-life
G6PD deficiency	4.6%	4.6%	4.6%
CYP2D6 slow metabolizers	3.4%	3.4%	3.4%
Pregnancy	7.5%	7.5%	7.5%
**Coverage**			
Case-management	100%	80%	80%
Intervention	80%	80%	80%
**Bloodstage drug**			
Efficacy (alone)	100%	100%	89.9%
Efficacy (with anti-hpz)	100%	100%	94.6%
Prophylaxis duration	28 days	28 days	14 days
**Hypnozoiticidal drug**			
Efficacy	100%	80%	71.36%
Prophylaxis duration	28 days	8 days	8 days
Adherence	100%	80%	66.7%
Minimum age	0 days	180 days	180 days
Pregnancy	administer	don't administer	don't administer
G6PD deficiency	administer	don't administer	don't administer
CYP2D6 slow metabolizers	effective	not effective	not effective

Public health impact, the outcome of interest in every simulation, was defined as the reduction of *P. vivax* prevalence measured by qPCR 6 months after the last round of Intervention and was calculated as $\frac{Prev.\kern0.5em before\ 1 st\ intervention- Prev.\kern0.5em at\ assessment\ period}{Prev.\kern0.5em before\ 1 st\ intervention}$. Overtreatment with a hypnozoiticidal drug was defined as the administration of the drug in a participant that had not been infected with *P. vivax* in the previous 9 months [18].

## Results

### Intervention-driven drop in qPCR prevalence

**Fig. 1:**
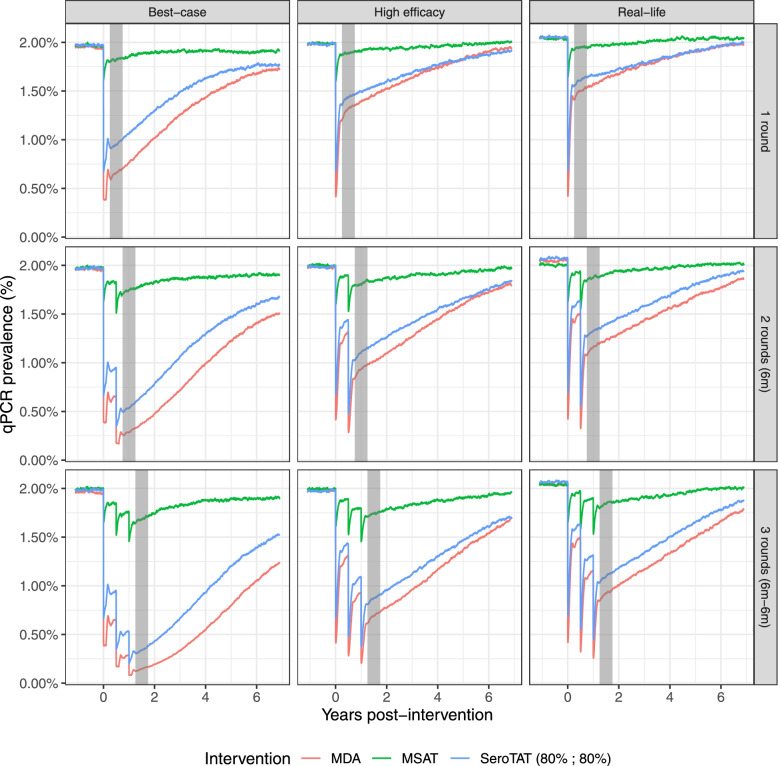
Modeled *P. vivax* qPCR prevalence with various implementations of public health interventions in an endemic situation of low transmission. The columns correspond to the different scenarios and rows to the number of intervention rounds, with gray area presenting time of evaluation

In the absence of sustained vector-control, the prevalence of *vivax* malaria is expected to rebound to its initial level after the last round of public health intervention is enforced, if elimination is not achieved. As such, the choice of an assessment window largely affects the measured impact, as can be seen in Fig. 1. In a moderate transmission setting, the prevalence is expected to rebound to its initial level within ~5 years, while in a low transmission setting, this duration is closer to ~8–10 years due to the slower rate of reinfection (Additional File 1: Figure S1). In all tested scenarios, MDA provided the highest impact as may be expected from a campaign enforcing radical cure of *P. vivax* population-wide, only excluding individuals because of 8-aminoquinole toxicity concerns. MSAT always resulted in the poorest impact, by systematically missing asymptomatic hypnozoite carriers at the time of screening. The reduction in *P.* vivax PCR prevalence caused by *Pv*SeroTAT was substantially greater than the reduction caused by MSAT, but less than the reduction caused by MDA.

### Upper and lower limits of achievable impact are defined by MDA and MSAT

Under ideal conditions (i.e., perfect clearance of hypnozoites, no safety concerns), 1, 2, or 3 rounds of MDA would yield prevalence reductions of respectively 65.8%, 85.6%, and 92.4% in a low transmission (2% PCR prevalence, Table 2) setting and 59.5%, 76.4%, and 84.1% in a moderate setting (10% PCR prevalence, Additional File 2: Table S2), respectively. However, MDA also results in overtreating 67.5–73.7% of the population in low transmission settings and 46.0–61.8% in moderate transmission settings. MDA campaigns with the “high efficacy” primaquine regimen are predicted to reduce PCR prevalence by 33.6–65.0% in low transmission areas and by 31.7–56.7% in moderate, with an overtreatment rate of 65.7–68.0% and 46.9–53.5%, respectively. In both scenarios, MDA impact and overtreatment rates were further reduced in the “real-life” primaquine scenario (Table 2 & Additional File 2: Table S2).

**Table 2:** Public health impact and overtreatment modeled in a low transmission setting (qPCR prevalence ~2%). Impact was defined as the reduction in qPCR prevalence observed 6 months after the last round of intervention; overtreatment corresponded to the administration of a hypnozoiticidal drug to a person whose last blood-stage infection occurred more than 9 months ago. The “Best-case,” “High efficacy,” and “Real-life” scenario correspond to those described in the main text, with decreasing adherence, efficacy, and eligibility

Intervention	Rounds	Sensitivity	Specificity	Impact (%)			Overtreatment (%)		
				*Best-case*	*High efficacy*	*Real-life*	*Best-case*	*High efficacy*	*Real-life*
MDA	1			65.8	33.6	27.1	67.5	65.7	66.3
MSAT	1			7.1	5.1	4.5	0	0	0
*Pv*SeroTAT	1	0.700	0.700	45.5	24.8	19.9	20.2	19.7	19.9
	1	0.800	0.800	51.9	27.8	21.4	13.5	13.2	13.3
	1	0.900	0.900	56.6	30.2	22.9	6.7	6.5	6.6
	1	1.000	1.000	61.9	32.3	25.7	0	0	0
	1	0.650	0.950	41.9	23.6	17.2	3.4	3.3	3.3
	1	0.950	0.650	60.6	31.1	23.9	23.7	23	23.2
MDA	2			85.6	52.9	43.5	69.2	66.1	66.7
MSAT	2			11.6	10	7.5	0	0	0
*Pv*SeroTAT	2	0.700	0.700	67.5	40.4	33.6	20.5	19.8	20
	2	0.800	0.800	73.1	44.6	36	13.7	13.2	13.3
	2	0.900	0.900	77.5	48.2	38.9	6.8	6.6	6.7
	2	1.000	1.000	81.9	51.6	41.2	0	0	0
	2	0.650	0.950	63.1	38.5	30.8	3.4	3.3	3.3
	2	0.950	0.650	81.8	49.4	40.6	24	23.2	23.3
MDA	3			92.4	65	54.7	73.7	68	68.3
MSAT	3			15.9	13.2	10.1	0	0	0
*Pv*SeroTAT	3	0.700	0.700	78.5	52	43.4	21.6	20.3	20.3
	3	0.800	0.800	82.9	55.8	46.8	14.5	13.5	13.5
	3	0.900	0.900	86.4	59.4	49.5	7.2	6.8	6.8
	3	1.000	1.000	89.8	62.9	52.6	0	0	0
	3	0.650	0.950	73.7	48.6	39.5	3.6	3.3	3.4
	3	0.950	0.650	89.6	61.3	52.5	25.6	23.8	23.9

MSAT with RDT had a substantially lower predicted impact on *P. vivax* transmission than MDA in every scenario as it failed to detect and cure the reservoir of asymptomatic, hypnozoite carriers (low transmission: “best-case” 7.1–15.9%, “high efficacy” 5.1–13.2%, and “real-life” 4.5–10.1%; moderate transmission: “best-case” 5.4–9.6%, “high efficacy” 3.3–7.1%, and “real-life” 2.9–6.5%). However, MSAT did not cause any overtreatment, as only individuals who had detectable blood-stage parasitemia were treated.

### PvSeroTAT as a safer and efficient alternative to MDA

Under the “best-case” implementation scenario, *Pv*SeroTAT with a *P. vivax* SDT of 80% sensitivity and 80% specificity is predicted to cause 51.9–82.9% in a low transmission setting (Table 2) and 45.9–71.7% reduction in *P. vivax* prevalence in a moderate setting (Additional File 2: Table S2). This equates to 77.1–89.7% of what would be achieved by implementing the corresponding 1, 2, or 3 rounds of MDA campaigns (Fig. 1 and Additional File 1: Figure S1). *Pv*SeroTAT similarly achieves 80.4–83.6% and 81.9–84.3% of the predicted MDA impact under the “high efficacy” and “real-life” scenarios (Tables 2 & Additional File 2: Table S2). These effects come with the benefit of a 5-fold reduction in overtreatment compared to MDA, only exposing 9.2–14.5% of the target population to unnecessary drugs (Tables 2 & Additional File 2: Table S2), in the moderate and low transmission settings, respectively.

### Relationship between diagnostic performance and PvSeroTAT impact and overtreatment

**Fig. 2:**
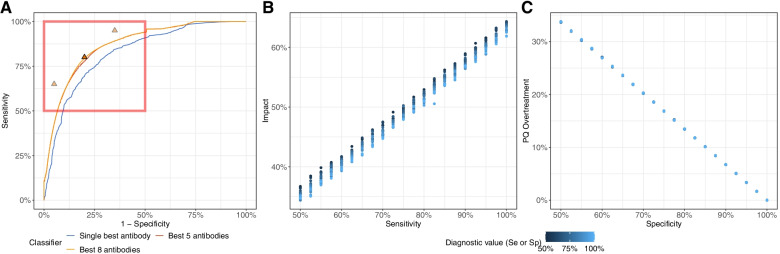
Exploration of the parameter space for the ROC curve of PvSeroTAT in a low transmission setting, with a single round of intervention using a hypothetical optimal hypnozoiticidal drug. **A** The red square on the full ROC curve represents the parameter space explored as part of the target product profiling. The three triangles correspond to a diagnostic test at 80% sensitivity and specificity (solid color; currently achieved by our antibody panel) as well as two alternatives at 65% and 90% (transparency; tradeoffs between sensitivity and specificity). **B** Association between impact (prevalence reduction after 6 months) and sensitivity. **C** Association between overtreatment and specificity. In panels **B** and **C**, dots are colored according to the diagnostic criteria not used on the *x*-axis

**Fig. 3:**
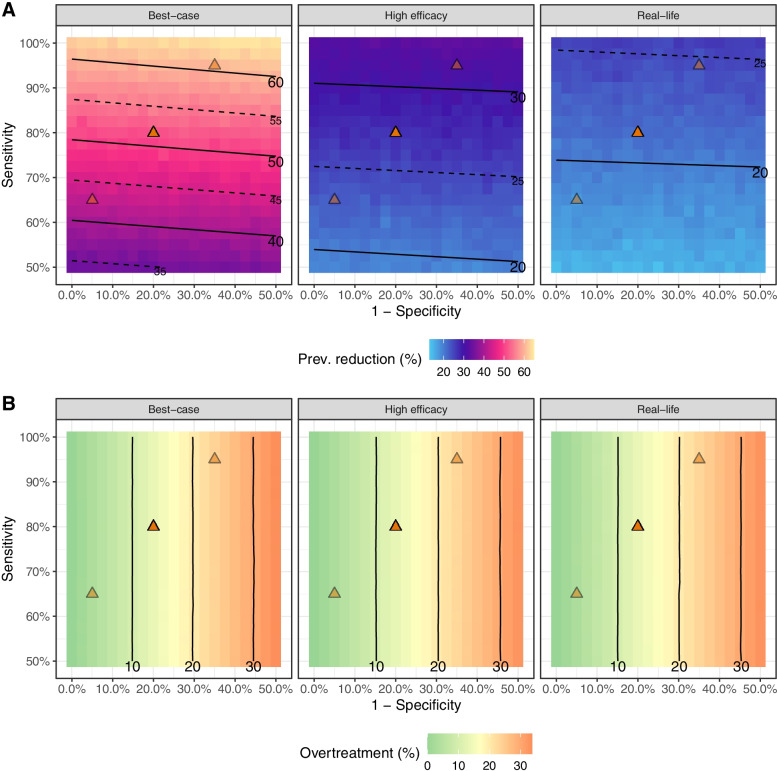
ROC surfaces for **A** impact and **B** overtreatment with a hypnozoiticidal drug under the different envisioned scenarios after a single round of *Pv*SeroTAT in a low transmission setting

The predicted impact of *Pv*SeroTAT on transmission was largely dependent on the sensitivity of the sero-diagnostic test, while the amount of overtreatment dispensed during rounds was directly proportional to the false positive rate (1 – specificity) of the sero-diagnostic test (see Additional Files 3, 4 and 5: Figures S3–S5). Under the “best-case” scenario the impact of a single-round *Pv*SeroTAT impact increased by about 5% for each 10% increase in diagnostic sensitivity (Fig. 2). The impact of *Pv*SeroTAT strategies varied substantially according to treatment scenarios (Fig. 3) but remained almost entirely linked to diagnostic sensitivity with impact rising from 24.8% and 19.9% for a 70% sensitivity & specificity test to 30.2% and 22.9% for 90% sensitivity & specificity test for a single round of *Pv*SeroTAT under the “high efficacy” and “real-life” scenarios for the low transmission settings (Table 2). For all diagnostic performance levels, *Pv*SeroTAT was predicted to have a greater effect size in the low transmission setting irrespective of the type of treatment or the number of *Pv*SeroTAT rounds administered (Table 2 and Additional File 2: Table S2, Fig. 3 and Additional File 1: Figure S1).

Overtreatment with hypnozoiticidal drugs is primarily the result of false positive diagnostics, that is, individuals who had higher antibody titers but did not have hypnozoites. The rate of overtreatment was not substantially affected by *Pv*SeroTAT performance or the number of *Pv*SeroTAT rounds administered and remained at approximately 5–7%, 10–14%, and 15–20% for 90%, 80%, and 70% diagnostic specificity tests in both transmission settings (Table 2 & Additional File 2: Table S2, Additional Files 3, 4 and 5: Figures S3–S5).

### Diagnostic performance and impact across multiple PvSeroTAT rounds

The impact of the *Pv*SeroTAT interventions increased with the number of rounds delivered in both transmission settings and all three implementation scenarios. For three rounds of *Pv*SeroTAT in the low transmission setting, impact rose to 78.5%, 82.9%, and 86.4% for 70/70, 80/80, and 90/90 sensitivity & specificity under the “best-case” treatment scenario, 52.0%, 55.8%, and 59.4% under the “high efficacy” scenario, and 43.4%, 46.8%, and 49.5% under the “real-life” scenario (Table 2). The relative improvement of impact of 3 vs 1 round of *Pv*SeroTAT was larger for “high efficacy” (80/80 +100%) and “real-life” (80/80 +119%) than the “best-case” scenario (80/80 +60%) and increased with decreasing assay performance (e.g., “high efficacy”: 70/70 +109%, 80/80: +100%, 90/90 +96%).

Even with increasing *Pv*SeroTAT rounds, the proportion of people overtreated remained constrained below 15% under the low transmission setting, with very low variations for the 80/80 test performance (relative increase vs. a single round: “best-case” +7%, “high efficacy” +3%, “real-life” +2%; Table 2). Similar trends, but with slightly smaller improvements in impact and even larger increases in overtreatment were predicted for the high transmission settings (Additional File 2: Table S2). However, the absolute number of people tested and to a lesser degree those treated however increased linearly with the number of *Pv*SeroTAT rounds.

## Discussion

Similar to previous observations from systematic reviews of field studies, our models predict that MDA programs have a strong but transient effect on *vivax* malaria transmission [23] that is estimated to be higher in low transmission settings [24, 25], but result in a large proportion of people receiving unnecessary treatments. In contrast with MDA, MSAT would result in poor impact in both transmission settings, but no overtreatment. This confirms the finding from a clinical trial in Indonesia [17] that treating only blood-stage *vivax* cases was not enough to lower the overall transmission.

*Pv*SeroTAT is a newly proposed public health intervention where community members are screened using an SDT and those classified as currently and previously exposed to *P. vivax* infections treated with anti-blood- and liver-stage therapy [18, 19]. *Pv*SeroTAT can bridge the gap between MDA and MSAT by achieving an impact on transmission that is close to that of MDA while dramatically reducing the level of overtreatment. Using a test with performance comparable to the currently available research assay (i.e., 80% sensitivity and 80% specificity [18]), *Pv*SeroTAT is predicted to achieve approximately 80–85% of the impact of MDA while reducing the number of people over-treated by 80%. The modeling of different combinations of SDT diagnostic performance showed that under both high and low transmission setting and all implementation scenarios the impact of 1 to 3 rounds of *Pv*SeroTAT impact is directly dependent on the sensitivity of the sero-diagnostic test (Table 2 & Additional File 2: Table S2, Fig. 3). The degree of overtreatment was however related entirely to test specificity.

This has important implications for *P. vivax* SDT development. Trade-offs between sensitivity and specificity during SDT development results in related trade-offs between public health impact and the rate of overtreatment. Yet, we found that multiple rounds of interventions with a lower sensitivity test may achieve the same impact as fewer rounds with a more sensitive one. For example, a public health campaign consisting of three rounds of *Pv*SeroTAT 6 months apart, at 70% sensitivity and 70% specificity would yield a public health impact comparable to that of two rounds at 90% sensitivity and 90% specificity (Tables 2 and 3). However, three rounds at 70/70 will result in more than four times as many people being unnecessarily treated compared to two rounds at 90/90. On the other hand, three rounds of *Pv*SeroTAT at 80% sensitivity and 80% specificity diagnostic performance is predicted to result in a similar impact to that achieved with two MDA rounds but accompanied by a 3-fold reduction in overtreatment.

As with other public health interventions, the impact is predicted to decrease as the implementation scenario moves from “best-case” to “real-life”. While results from the ideal scenario provide a higher boundary of treatment efficacy (i.e., universal eligibility for hypnozoiticidal drug treatment), the differences in impact observed between the “high efficacy” and the “real-life” scenario are attributable to decreased efficacy and adherence to primaquine regimens. This highlights the importance of strong locally led programmatic implementation and the need for effective communication and engagement with treated populations. Shorter treatment with primaquine at increased dosage, such as that used in the IMPROV study [11] or a single dose of tafenoquine may also provide increased adherence. Both the use of a *P. vivax* SDT with higher sensitivity or an increase in the number of *Pv*SeroTAT rounds delivered can at least partially counteract the impact decays inherent in “real-life” programmatic implementation. In a recent Phase III clinical trial, tafenoquine demonstrated non-inferiority with respect to low-dose Primaquine regimen to prevent hypnozoite-caused relapses. Therefore, although we did not model explicitly Tafenoquine due to it not being readily available in many endemic regions, we expect its efficacy to be similar to what was observed in our “high efficacy” scenario.

A key output from our model was that *Pv*SeroTAT alone was not enough to reach elimination either in low or moderate transmission settings. Rather than modeling elimination scenarios, we set out to estimate the expected magnitude of reduction in PCR prevalence upon implementing a population-level public health campaign. We propose *Pv*SeroTAT to be used as a tool with high potential for temporarily shrinking the human parasite reservoir, jointly with tools such as vector control or other measures targeting residual pockets of transmission (e.g., reactive case-detection, RCD) and vector populations. In low-transmission settings, some countries have started to implement RCD to focus interventions around newly identified index cases. A notable example is the 1–3–7 surveillance and response strategy developed in China with the aim to report new cases within 1 day, investigate these within 3 days, and take action within 7 days [26–28]. As part of these actions, *Pv*SeroTAT could be used to improve upon microscopy-based detection methods (i.e., MSAT) upon investigating possible asymptomatic infections linked to the index case. A limitation of our model lies in the absence of geographically-explicit enhanced actions to be taken in the vicinity of infections treated by the routine case-management health system.

In our modeling approach, overtreatment was defined as administering hypnozoiticidal drugs to an individual without a blood-stage infection in the previous 9 months. Most *P. vivax* relapses are expected to occur within 9 months of the primary infection [29]. Overtreatment is therefore the result of false positive diagnostics (i.e., blood-stage infection happened more than 9 months previously). A different definition of overtreatment, based on presence or absence of hypnozoites, would result in somewhat different estimates of overtreatment as the antibody levels may indicate a positive diagnostic signal for individuals who had a blood-stage infection in the last 9 months but do not harbor hypnozoites anymore.

A limitation of our analysis is that we do not account for potential variation between geographic regions and across transmission intensities, factors which are known to affect diagnostic accuracy [30]. Another limitation of this analysis is that we did not account for the effects of targeting diagnostics and treatment strategies at high-risk populations. For example, in Cambodia, malaria transmission was found to be mostly associated with occupational activities such as forestry or living close to forested areas [31]. The effectiveness of *Pv*SeroTAT interventions could be optimized by targeting people who are at increased risk of malaria infection, for example when transmission is mostly occupational. Furthermore, malaria transmission fluctuates seasonally and we did not set out to investigate the timing of interventions. Further studies will assess the impact of single or multiple rounds happening either during low or peak transmission season, so that public health campaigns can be enforced at the time where they will most reduce *P. vivax* transmission.

## Conclusion

Current antibody panels allow for detecting individuals who are at a high risk of *P. vivax* relapse with a diagnostic performance of approximately 80% sensitivity and 80% specificity. We predict that *Pv*SeroTAT campaigns may achieve an impact similar to that of MDAs, with the benefit of massively reducing overexposure to primaquine, thus lowering the risks of G6PDd-induced haemolysis. To achieve effective *Pv*SeroTAT implementation, the development of *P. vivax* SDTs should aim for both high performance and easy field-use thus facilitating deployment of multiple *Pv*SeroTAT rounds. Further cost-impact modeling will be required to determine if a more expensive but higher performing test may have a better cost-effectiveness than a lower performing test that requires an additional *Pv*SeroTAT round to achieve the same public health impact. We suggest that with a field-deployable test achieving a diagnostic performance of the current research assay (i.e., 80% sensitivity and 80% sensitivity [18], *Pv*SeroTAT would provide sufficient public-health impact for programmatic implementation in countries that have already achieved a reduction in transmission levels to low-to-moderate levels (2–10% *P. vivax* prevalence by qPCR), where highest *Pv*SeroTAT impact is expected. The target product profiles informing *P. vivax* SDT development should reflect the trade-offs between impact, overtreatment, and ease of programmatic implementation that are identified here.

## Supplementary Information

**Additional file 1: Figure S1.** Modeled *P. vivax* qPCR prevalence with various implementations of public health interventions in an endemic situation of moderate transmission. The columns correspond to the different scenarios and rows to the number of intervention rounds, with grey area presenting time of evaluation.

**Additional file 2: Table S2.** Public health impact and overtreatment modeled in a moderate transmission setting (qPCR prevalence ~10%). Impact was defined as the reduction in qPCR prevalence observed six months after the last round of intervention; overtreatment corresponded to the administration of a hypnozoiticidal drug to a person whose last blood-stage infection occurred more than 9 months ago. The “Best-case”, “High efficacy” and “Real-life” scenario correspond to those described in the main text, with decreasing adherence, efficacy and eligibility.

**Additional file 3: Figure S3.** Heat maps for (A) impact and (B) overtreatment with an optimal hypnozoiticidal drug as defined by the “best-case” scenario and after 1, 2 or 3 rounds of *Pv*SeroTAT and under two transmission pressures.

**Additional file 4: Figure S4.** ROC surfaces for (A) impact and (B) overtreatment with Primaquine administered in a high-efficacy scenario after 1, 2 or 3 rounds of *Pv*SeroTAT and under two transmission pressures.

**Additional file 5: Figure S5.** Heat maps for (A) impact and (B) overtreatment with Primaquine in a real-life scenario after 1, 2 or 3 rounds of *Pv*SeroTAT and under two transmission pressures.

## Data Availability

The model developments and C++ code are hosted on a GitLab repository at https://gitlab.pasteur.fr/mwhite/pv_mod. The model used to run all simulations corresponds to commit 85620fedb494b5f99351c4d876d6904665e579ce found in the master branch of that repository.

## Abbreviations

MDA: Mass drug administration
MSAT: Mass screen-and-treat
*Pv*SeroTAT: *Plasmodium vivax* serological test-and-treat
RDT: Rapid diagnostic test
G6PDd: Glucose-6-phosphate dehydrogenase deficiency
CYP2D6: Cytochrome P450 2D6
TPP: Target product profile
SDT: Sero-diagnostic test
WHO: World Health Organization
qPCR: Quantitative polymerase chain reaction
