# Supplementary material for: Developing sero-diagnostic tests to facilitate Plasmodium vivax Serological Test-and-Treat approaches: modeling the balance between public health impact and overtreatment
Source: BMC Med. 2022 Mar 18;20:98. doi: 10.1186/s12916-022-02285-5 (PMC8932240; doi:10.1186/s12916-022-02285-5)
Supplement: Supplementary file 1 — Additional file 1: Figure S1. Modeled P. vivax qPCR prevalence with various implementations of public health interventions in an endemic situation of moderate transmission. The columns correspond to the different scenarios and rows to the number of intervention rounds, with grey area presenting time of evaluation. [file 12916_2022_2285_MOESM1_ESM.docx]

**Additional File 1**

**Figure S1. Modeled *P. vivax* qPCR prevalence with various implementations of public health interventions in an endemic situation of moderate transmission.** The columns correspond to the different scenarios and rows to the number of intervention rounds, with grey area presenting time of evaluation.
