# Supplementary material for: Developing sero-diagnostic tests to facilitate Plasmodium vivax Serological Test-and-Treat approaches: modeling the balance between public health impact and overtreatment
Source: BMC Med. 2022 Mar 18;20:98. doi: 10.1186/s12916-022-02285-5 (PMC8932240; doi:10.1186/s12916-022-02285-5)
Supplement: Supplementary file 2 — Additional file 2: Table S2. Public health impact and overtreatment modeled in a moderate transmission setting (qPCR prevalence ~10%). Impact was defined as the reduction in qPCR prevalence observed six months after the last round of intervention; overtreatment corresponded to the administration of a hypnozoiticidal drug to a person whose last blood-stage infection occurred more than 9 months ago. The “Best-case”, “High efficacy” and “Real-life” scenario correspond to those described in the main text, with decreasing adherence, efficacy and eligibility. [file 12916_2022_2285_MOESM2_ESM.docx]

**Additional File 2**

**Table S2. Public health impact and overtreatment modeled in a moderate transmission setting (qPCR prevalence ~10%).** Impact was defined as the reduction in qPCR prevalence observed six months after the last round of intervention; overtreatment corresponded to the administration of a hypnozoiticidal drug to a person whose last blood-stage infection occurred more than 9 months ago. The “Best-case”, “High efficacy” and “Real-life” scenario correspond to those described in the main text, with decreasing adherence, efficacy and eligibility.

| **Intervention** | **Rounds** | **Sensitivity** | **Specificity** | **Impact (%)** | | | **Overtreatment (%)** | | | |  |
| --- | --- | --- | --- | --- | --- | --- | --- | --- | --- | --- | --- |
|  |  |  |  | *Best-case* | *High efficacy* | *Real-life* | | *Best-case* | *High efficacy* | *Real-life* | |
| MDA | 1 |  |  | 59.5 | 31.7 | 23.8 | | 46 | 46.9 | 48 | |
| MSAT | 1 |  |  | 5.4 | 3.3 | 2.9 | | 0 | 0 | 0 | |
| *Pv*SeroTAT | 1 | 0.700 | 0.700 | 40.5 | 22.8 | 17 | | 13.8 | 14 | 14.4 | |
|  | 1 | 0.800 | 0.800 | 45.9 | 25.5 | 19.5 | | 9.2 | 9.4 | 9.6 | |
|  | 1 | 0.900 | 0.900 | 52.1 | 28.3 | 21.3 | | 4.6 | 4.7 | 4.8 | |
|  | 1 | 1.000 | 1.000 | 57.6 | 30.7 | 23.6 | | 0 | 0 | 0 | |
|  | 1 | 0.650 | 0.950 | 37.2 | 21.2 | 16.4 | | 2.3 | 2.3 | 2.4 | |
|  | 1 | 0.950 | 0.650 | 55.6 | 30 | 22.9 | | 16.1 | 16.4 | 16.8 | |
| MDA | 2 |  |  | 76.4 | 47.5 | 37.5 | | 49.8 | 48 | 49 | |
| MSAT | 2 |  |  | 8 | 5.5 | 5 | | 0 | 0 | 0 | |
| *Pv*SeroTAT | 2 | 0.700 | 0.700 | 56.9 | 35.6 | 27.7 | | 14.5 | 14.3 | 14.6 | |
|  | 2 | 0.800 | 0.800 | 63.6 | 39.1 | 30.8 | | 9.6 | 9.6 | 9.7 | |
|  | 2 | 0.900 | 0.900 | 69.7 | 43.2 | 34.5 | | 4.8 | 4.8 | 4.9 | |
|  | 2 | 1.000 | 1.000 | 75.1 | 46.5 | 37 | | 0 | 0 | 0 | |
|  | 2 | 0.650 | 0.950 | 53.6 | 33.6 | 26.6 | | 2.4 | 2.4 | 2.4 | |
|  | 2 | 0.950 | 0.650 | 72.8 | 45.5 | 35.8 | | 17.1 | 16.8 | 17.1 | |
| MDA | 3 |  |  | 84.1 | 56.7 | 45.9 | | 61.8 | 53.5 | 53.3 | |
| MSAT | 3 |  |  | 9.6 | 7.1 | 6.5 | | 0 | 0 | 0 | |
| *Pv*SeroTAT | 3 | 0.700 | 0.700 | 65.2 | 43.4 | 35 | | 16.9 | 15.4 | 15.5 | |
|  | 3 | 0.800 | 0.800 | 71.7 | 47.4 | 38.7 | | 11.5 | 10.4 | 10.4 | |
|  | 3 | 0.900 | 0.900 | 77.3 | 52.1 | 42.6 | | 5.9 | 5.2 | 5.2 | |
|  | 3 | 1.000 | 1.000 | 81.6 | 55.9 | 45.7 | | 0 | 0 | 0 | |
|  | 3 | 0.650 | 0.950 | 61.6 | 41.1 | 33.4 | | 2.8 | 2.5 | 2.6 | |
|  | 3 | 0.950 | 0.650 | 80.3 | 54.3 | 44 | | 21.1 | 18.6 | 18.5 | |
