# Supplementary material for: Developing sero-diagnostic tests to facilitate Plasmodium vivax Serological Test-and-Treat approaches: modeling the balance between public health impact and overtreatment
Source: BMC Med. 2022 Mar 18;20:98. doi: 10.1186/s12916-022-02285-5 (PMC8932240; doi:10.1186/s12916-022-02285-5)
Supplement: Supplementary file 5 — Additional file 5: Figure S5. Heat maps for (A) impact and (B) overtreatment with Primaquine in a real-life scenario after 1, 2 or 3 rounds of PvSeroTAT and under two transmission pressures. [file 12916_2022_2285_MOESM5_ESM.docx]

**Additional File 5**

**Figure S5. Heat maps for (A) impact and (B) overtreatment with Primaquine in a real-life scenario after 1, 2 or 3 rounds of *Pv*SeroTAT and under two transmission pressures.**
